# Real-world effectiveness of palbociclib plus an aromatase inhibitor in HR+/HER2− MBC patients living in disadvantaged neighborhoods

**DOI:** 10.1038/s41523-025-00786-z

**Published:** 2025-07-21

**Authors:** Filipa Lynce, Xianchen Liu, Benjamin Li, Lynn McRoy, Connie Chen, Raymond Liu, Hope S. Rugo

**Affiliations:** 1 Department of Medical Oncology, Dana-Farber Cancer Institute, Boston, MA USA; 2 Harvard Medical School, Boston, MA USA; 3 Pfizer Inc., New York, NY USA; 4 Division of Research, Kaiser Permanente Northern California, Oakland, CA USA; 5 San Francisco Medical Center, Kaiser Permanente Northern California, San Francisco, CA USA; 6 Department of Medical Oncology & Therapeutics Research, City of Hope Comprehensive Cancer Center, Duarte, CA USA

**Keywords:** Breast cancer, Health care economics

## Abstract

Palbociclib (PAL) combined with endocrine therapy (ET) is approved for hormone receptor-positive/human epidermal growth factor receptor 2-negative (HR+/HER2− ) advanced/metastatic breast cancer (MBC). However, there is limited data on the effectiveness of PAL + ET in patients with MBC and low socioeconomic status. This retrospective study of the Flatiron Health database compared overall survival (OS) and real-world progression-free survival (rwPFS) in patients with MBC living in disadvantaged neighborhoods who received either first-line PAL with an aromatase inhibitor (AI) or an AI alone. Of 723 patients, 394 received PAL + AI and 329 received AI alone. After stabilized inverse probability of treatment weighting, median OS was 57.1 months versus 38.2 months (hazard ratio, 0.70, *P* = 0.0053) and median rwPFS was 19.1 months versus 14.0 months (hazard ratio, 0.66, *P* = 0.0007) for PAL + AI versus AI alone, respectively. This real-world data analysis demonstrated that first-line PAL + AI versus AI alone was associated with survival benefit in patients with HR+/HER2− MBC living in disadvantaged neighborhoods. Trial registration number: NCT06495164.

## Introduction

Palbociclib, the first-in-class oral cyclin-dependent kinase 4/6 (CDK4/6) inhibitor, was initially approved in the United States in combination with endocrine therapy (ET) for the treatment of hormone receptor-positive/human epidermal growth factor receptor 2-negative (HR+/HER2−) advanced/metastatic breast cancer (MBC) in February 2015^1^. Randomized controlled trials (RCTs) have demonstrated that first-line palbociclib in combination with AI versus placebo plus AI significantly prolonged progression-free survival (PFS, primary endpoint)^2,3^; overall survival (OS, secondary endpoint) was numerically prolonged across most subgroups, but did not reach statistical significance^4^.

A growing body of real-world evidence has demonstrated that palbociclib plus ET is more effective than ET alone in various populations of patients with HR+/HER2− MBC^5–10^. In a large (n = 2888), heterogeneous population in the United States, palbociclib plus ET was associated with a 30% reduction in risk of disease progression and a 24% reduction in risk of death relative to ET alone^8^. Real-world data sets have outcomes from all patients in routine clinical practice including patients who are often excluded from RCTs, and have been increasingly recognized as important complementary data sources that supplement evidence from RCTs^11,12^. Real-world evidence has demonstrated that patients aged ≥ 65 years and ≥ 75 years and those with metastases to the lungs and/or liver achieve a greater clinical benefit with palbociclib plus ET than with ET alone^5,6,10^. In African Americans with HR+/HER2− MBC, a population historically underrepresented in RCTs, relative risk reduction of progression (26%) and death (44%) were observed with palbociclib plus ET versus ET alone^9^. Despite this demonstration of palbociclib effectiveness in diverse patient subgroups, there is evidence that palbociclib and other CDK4/6 inhibitors have not been deployed as frequently as would be expected for a standard of care^13,14^.

There exist well-documented disparities in outcomes among racial and ethnic groups of patients with breast cancer^15,16^, including those with MBC^17^. A multitude of demographic, tumor, and other disease-related factors likely contribute to these inequalities, but social determinants of health (SDOH)^18^ have emerged as important predictors of breast cancer survival^17,19–21^. Using Surveillance, Epidemiology, and End Results (SEER) registry data, it was shown that patients residing in the most disadvantaged neighborhoods (bottom quintile) had a 43% greater risk of breast cancer-related death than those in the most advantaged neighborhoods (top quintile)^19^. Another SEER analysis was able to attribute approximately half of the excess risk for breast cancer-specific death experienced by non-Hispanic Black women with MBC aged < 65 years relative to their non-Hispanic White peers to socioeconomic factors^17^. Poorer outcomes among groups with unmet social needs may be linked to differential breast cancer management, as evidenced by longer time to treatment initiation^21^ and suboptimal treatment selection^13^.

Generating more equitable outcomes for patients with MBC in vulnerable populations such as those residing in low-income areas is a crucial public health objective. However, data regarding the use and effectiveness of CDK4/6 inhibitor/ET combination in patients with MBC living in disadvantaged neighborhoods are lacking; such findings may help to guide treatment decision-making by clinicians serving patient populations with SDOH-related barriers to care. Therefore, the current study aimed to compare OS and real-world PFS (rwPFS) with first-line palbociclib plus an AI versus an AI alone in patients with HR+/HER2− MBC living in disadvantaged neighborhoods in the United States.

## Results

### Patients

**Fig. 1:**
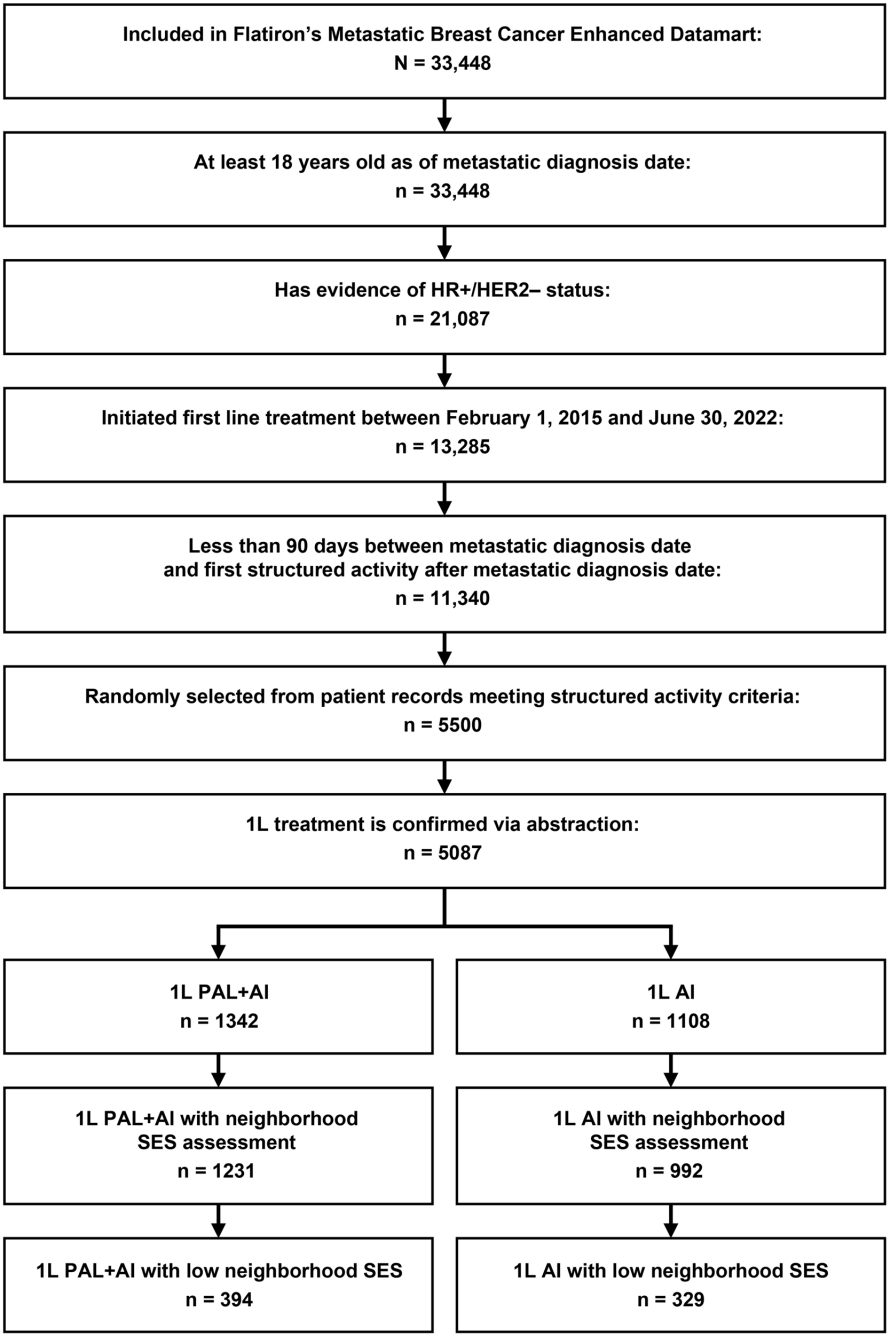
Patient attrition diagram. 1L first-line, AI aromatase inhibitor, HR+/HER2− hormone receptor-positive/human epidermal growth factor receptor 2-negative, PAL palbociclib, SES socioeconomic status.

Of the 5087 patients with HR+/HER2− MBC, 1342 received palbociclib plus an AI and 1108 received an AI alone as first-line therapy. Of 1342 patients who received palbociclib plus an AI, 1231 (91.7%) had neighborhood socioeconomic status (SES) assessments and 394 (29.4%) met the criteria for living in disadvantaged neighborhoods. Of 1108 patients who received an AI alone, 992 (89.5%) had neighborhood SES assessments and 329 (29.7%) met the criteria for living in disadvantaged neighborhoods (Fig. 1). Median follow-up duration was 27.2 months for palbociclib plus an AI and 25.7 months for patients treated with an AI alone. Compared with the AI alone group, the palbociclib plus AI group was younger (median age 66.0 versus 69.0 years) and had higher proportions of de novo MBC (39.8% versus 24.0%) and visceral metastasis (32.0% versus 19.8%; Table 1). After stabilized inverse probability of treatment weighting (sIPTW) and 1:1 propensity score matching (PSM), baseline demographic and clinical characteristics were generally well balanced between the palbociclib plus AI and AI alone groups.

**Table 1:** Patient demographics and disease characteristics

Characteristic	Unadjusted analysis			sIPTW analysis			PSM analysis		
	1 L PAL + AI (*n* = 394)	1 L AI alone (*n* = 329)	Standardized difference	1 L PAL + AI (*n* = 405)	1 L AI alone (*n* = 324)	Standardized difference	1 L PAL + AI (*n* = 186)	1 L AI alone (*n* = 186)	Standardized difference
**Age at MBC diagnosis, years**									
Mean (SD)	66.0 (11.0)	68.8 (10.9)	−0.2485	67.5 (11.5)	67.7 (10.6)	−0.0195	67.9 (11.5)	68.5 (11.0)	−0.0497
Median (IQR)	66 (16)	69 (17)	–	69 (18)	68 (16)	–	70 (19)	69 (17)	–
**Age group,**^**a**^ **years,** ***n*** (%)									
18−49	25 (6.3)	16 (4.9)	0.0645	21 (5.3)	19 (5.8)	−0.0216	11 (5.9)	10 (5.4)	0.0233
50−64	147 (37.3)	103 (31.3)	0.1267	137 (34.0)	110 (33.8)	0.0026	53 (28.5)	61 (32.8)	−0.0934
65−74	128 (32.5)	95 (28.9)	0.0784	120 (29.6)	102 (31.5)	−0.0410	61 (32.8)	55 (29.6)	0.0697
75+	94 (23.9)	115 (35.0)	−0.2454	126 (31.1)	94 (28.9)	0.0493	61 (32.8)	60 (32.3)	0.0115
**Sex,**^**a**^ ***n*** **(%)**									
Male	4 (1.0)	3 (0.9)	0.0106	4 (1.1)	4 (1.3)	−0.0150	2 (1.1)	1 (0.5)	0.0601
Female	390 (99.0)	326 (99.1)	–	400 (98.9)	320 (98.7)	–	184 (98.9)	185 (99.5)	–
**Race,**^**a**^ ***n*** **(%)**									
White	236 (59.9)	189 (57.4)	0.0498	230 (56.8)	190 (58.6)	−0.0376	108 (58.1)	110 (59.1)	−0.0218
Black	67 (17.0)	66 (20.1)	−0.0787	70 (17.2)	57 (17.5)	−0.0079	35 (18.8)	32 (17.2)	0.0420
Other^b^	91 (23.1)	74 (22.5)	0.0144	105 (26.0)	77 (23.9)	0.0500	43 (23.1)	44 (23.7)	−0.0127
**Practice type,**^**a**^ ***n*** **(%)**									
Academic	68 (17.3)	44 (13.4)	0.1080	64 (15.8)	53 (16.4)	−0.0150	25 (13.4)	34 (18.3)	−0.1327
Community	326 (82.7)	285 (86.6)	–	341 (84.2)	271 (83.6)	–	161 (86.6)	152 (81.7)	–
**Insurance,** ***n*** **(%)**									
Commercial health plan + any other	135 (34.3)	115 (35.0)	−0.0145	135 (33.5)	113 (34.8)	−0.0288	69 (37.1)	67 (36.0)	0.0223
Commercial health plan	137 (34.8)	93 (28.3)	0.1403	145 (35.9)	91 (28.0)	0.1695	56 (30.1)	50 (26.9)	0.0715
Medicare	24 (6.1)	19 (5.8)	0.0134	24 (6.0)	21 (6.3)	−0.0137	11 (5.9)	13 (7.0)	−0.0438
Medicaid	11 (2.8)	8 (2.4)	0.0226	14 (3.5)	7 (2.2)	0.0762	6 (3.2)	6 (3.2)	0.0000
Other payer type	87 (22.1)	94 (28.6)	−0.1497	86 (21.1)	93 (28.6)	−0.1730	44 (23.7)	50 (26.9)	−0.0743
**Disease stage at initial diagnosis,**^**a**^ ***n*** **(%)**									
I	36 (9.1)	49 (14.9)	−0.1777	61 (15.0)	43 (13.3)	0.0493	22 (11.8)	28 (15.1)	−0.0947
II	111 (28.2)	95 (28.9)	−0.0156	116 (28.8)	91 (28.2)	0.0122	60 (32.3)	55 (29.6)	0.0582
III	61 (15.5)	68 (20.7)	−0.1351	65 (16.1)	58 (18.0)	−0.0499	32 (17.2)	29 (15.6)	0.0436
IV	157 (39.8)	79 (24.0)	0.3447	127 (31.4)	101 (31.0)	0.0094	58 (31.2)	58 (31.2)	0.0000
Not documented	29 (7.4)	38 (11.6)	−0.1436	35 (8.7)	31 (9.6)	−0.0287	14 (7.5)	16 (8.6)	−0.0395
**ECOG performance status,**^**a**^ ***n*** **(%)**									
0	146 (37.1)	73 (22.2)	0.3300	118 (29.1)	96 (29.6)	−0.0105	43 (23.1)	51 (27.4)	−0.0991
1	107 (27.2)	90 (27.4)	−0.0045	108 (26.7)	88 (27.2)	−0.0106	54 (29.0)	57 (30.6)	−0.0353
≥2	53 (13.5)	42 (12.8)	0.0203	51 (12.6)	42 (13.0)	−0.0115	30 (16.1)	26 (14.0)	0.0602
Not documented	88 (22.3)	124 (37.7)	−0.3398	128 (31.5)	98 (30.2)	0.0289	59 (31.7)	52 (28.0)	0.0823
**Visceral metastasis,**^**a,c**^ ***n*** **(%)**									
No	268 (68.0)	264 (80.2)	−0.2819	302 (74.6)	246 (75.7)	−0.0264	143 (76.9)	148 (79.6)	−0.0652
Yes	126 (32.0)	65 (19.8)	–	103 (25.4)	79 (24.3)	–	43 (23.1)	38 (20.4)	–
**Bone metastasis,**^**a**^ ***n*** **(%)**									
No	232 (58.9)	188 (57.1)	0.0353	242 (59.7)	189 (58.2)	0.0305	98 (52.7)	95 (51.1)	0.0323
Yes	162 (41.1)	141 (42.9)	–	163 (40.3)	136 (41.8)	–	88 (47.3)	91 (48.9)	–
**Brain metastasis,** ***n*** **(%)**									
No	388 (98.5)	323 (98.2)	0.0235	399 (98.7)	317 (97.7)	0.0756	183 (98.4)	182 (97.8)	0.0396
Yes	6 (1.5)	6 (1.8)	–	5 (1.3)	8 (2.3)	–	3 (1.6)	4 (2.2)	–
**No. of metastatic sites,**^**a,d**^ ***n*** **(%)**									
1	204 (51.8)	180 (54.7)	−0.0588	211 (52.1)	174 (53.7)	−0.0329	105 (56.5)	114 (61.3)	−0.0985
2	101 (25.6)	58 (17.6)	0.1954	84 (20.8)	67 (20.6)	0.0047	42 (22.6)	34 (18.3)	0.1068
3	38 (9.6)	17 (5.2)	0.1716	29 (7.3)	30 (9.1)	−0.0681	14 (7.5)	11 (5.9)	0.0645
4	22 (5.6)	8 (2.4)	0.1612	17 (4.3)	12 (3.6)	0.0372	7 (3.8)	6 (3.2)	0.0293
≥5	9 (2.3)	4 (1.2)	0.0816	7 (1.7)	4 (1.3)	0.0269	5 (2.7)	2 (1.1)	0.1189
Not documented	20 (5.1)	62 (18.8)	−0.4342	56 (13.9)	38 (11.6)	0.0679	13 (7.0)	19 (10.2)	−0.1152
**Disease-free interval in years,**^**a,e**^ ***n*** **(%)**									
De novo	157 (39.8)	79 (24.0)	0.3447	127 (31.4)	101 (31.0)	0.0094	58 (31.2)	58 (31.2)	0.0000
≤1	18 (4.6)	16 (4.9)	−0.0139	20 (4.8)	17 (5.3)	−0.0215	7 (3.8)	10 (5.4)	−0.0773
>1 − 5	64 (16.2)	110 (33.4)	−0.4060	107 (26.4)	80 (24.8)	0.0379	45 (24.2)	42 (22.6)	0.0381
>5	155 (39.3)	122 (37.1)	0.0465	151 (37.3)	125 (38.6)	−0.0275	76 (40.9)	76 (40.9)	0.0000
Not documented	0	2 (0.6)	−0.1106	0	1 (0.3)	−0.0750	0	0	–
**NCI Comorbidity Index**									
Mean (SD)	0.33 (0.5)	0.43 (0.6)	−0.1826	0.37 (0.5)	0.40 (0.5)	−0.0570	0.43 (0.6)	0.42 (0.6)	0.0151
**Follow-up duration, months**									
Median (IQR)	27.2 (32.3)	25.7 (43.4)	–	25.1 (32.2)	26.9 (45.6)	–	27.8 (33.4)	25.0 (40.1)	–

^a^Variable used in propensity score estimation; ^b^Other also includes Asian, Hispanic or Latino; ^c^Visceral disease was defined as metastatic disease in the lung and/or liver; ^d^Multiple metastases at the same site were counted as 1 site (e.g., if a patient had 3 bone metastases in the spine, it was considered only 1 site); ^e^Defined as the interval from initial breast cancer diagnosis to MBC diagnosis.

– no value, *1L* first-line; *AI* aromatase inhibitor, *ECOG* Eastern Cooperative Oncology Group, *IQR* interquartile range, *MBC* metastatic breast cancer, *NCI* National Cancer Institute, *No* number, *PAL* palbociclib, *PSM* propensity score matching, *SD* standard deviation, *sIPTW* stabilized inverse probability of treatment weighting.

### Overall survival

**Fig. 2:**
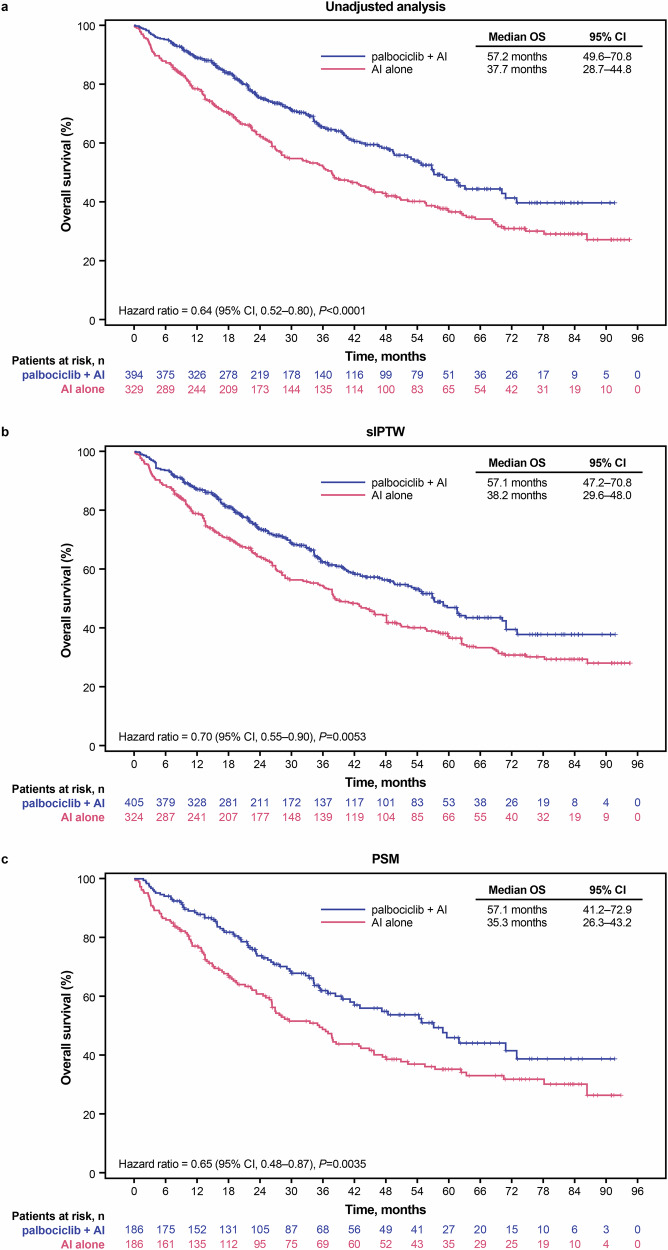
Kaplan–Meier curves of overall survival. Graphical representation of OS in **a** the unadjusted population, **b** following sIPTW, and **c** following PSM. AI aromatase inhibitor, CI confidence interval, OS overall survival, PAL palbociclib, PSM propensity score matching, sIPTW stabilized inverse probability of treatment weighting.

In the unadjusted analysis, patients from disadvantaged neighborhoods treated with palbociclib plus AI had significantly longer median OS than patients treated with AI alone (57.2 months [95% confidence interval (CI), 49.6–70.8] versus 37.7 months [95% CI, 28.7–44.8]; hazard ratio, 0.64 [95% CI, 0.52–0.80]; *P* < 0.0001; Fig. 2a). After sIPTW, palbociclib plus AI was associated with significantly longer OS versus treatment with AI alone (57.1 months [95% CI, 47.2–70.8] versus 38.2 months [95% CI, 29.6–48.0]; hazard ratio, 0.70 [95% CI, 0.55–0.90]; *P* = 0.0053; Fig. 2b). Similar results were seen in the PSM sensitivity analysis (57.1 months [95% CI, 41.2–72.9] versus 35.3 months [95% CI, 26.3–43.2]; hazard ratio, 0.65 [95% CI, 0.48–0.87]; *P* = 0.0035; Fig. 2c).

### Real-world PFS and PFS2

**Fig. 3:**
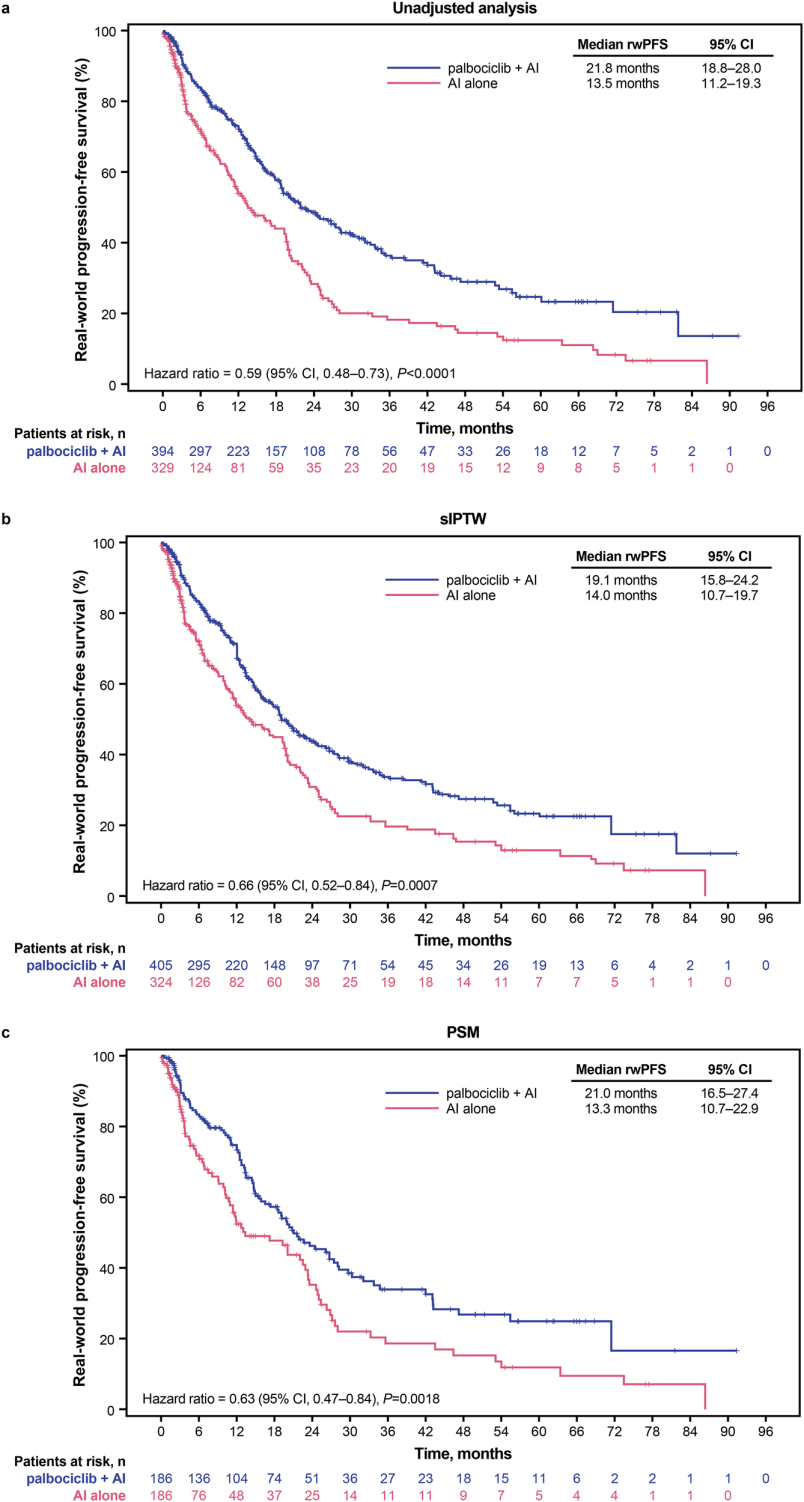
Kaplan–Meier curves of real-world progression-free survival. Graphical representation of rwPFS in **a** the unadjusted population, **b** following sIPTW, and **c** following PSM. AI aromatase inhibitor, CI confidence interval, PAL palbociclib, PSM propensity score matching, rwPFS real-world progression-free survival, sIPTW stabilized inverse probability of treatment weighting.

In the unadjusted analysis, patients from disadvantaged neighborhoods who were treated with palbociclib plus AI had median rwPFS of 21.8 months (95% CI, 18.8–28.0) compared with patients treated with AI alone (13.5 months [95% CI, 11.2–19.3]; hazard ratio, 0.59 [95% CI, 0.48–0.73]; *P* < 0.0001; Fig. 3a). After sIPTW, median rwPFS was 19.1 months (95% CI, 15.8–24.2) in the palbociclib plus AI-treated group versus 14.0 months (95% CI, 10.7–19.7) in the AI-treated group (hazard ratio, 0.66 [95% CI, 0.52–0.84]; *P* = 0.0007; Fig. 3b). Median rwPFS after PSM was 21.0 months (95% CI, 16.5–27.4) in the palbociclib plus AI group versus 13.3 months (95% CI, 10.7–22.9) in the AI alone group (hazard ratio, 0.63 [95% CI, 0.47–0.84]; *P* = 0.0018; Fig. 3c).

**Fig. 4:**
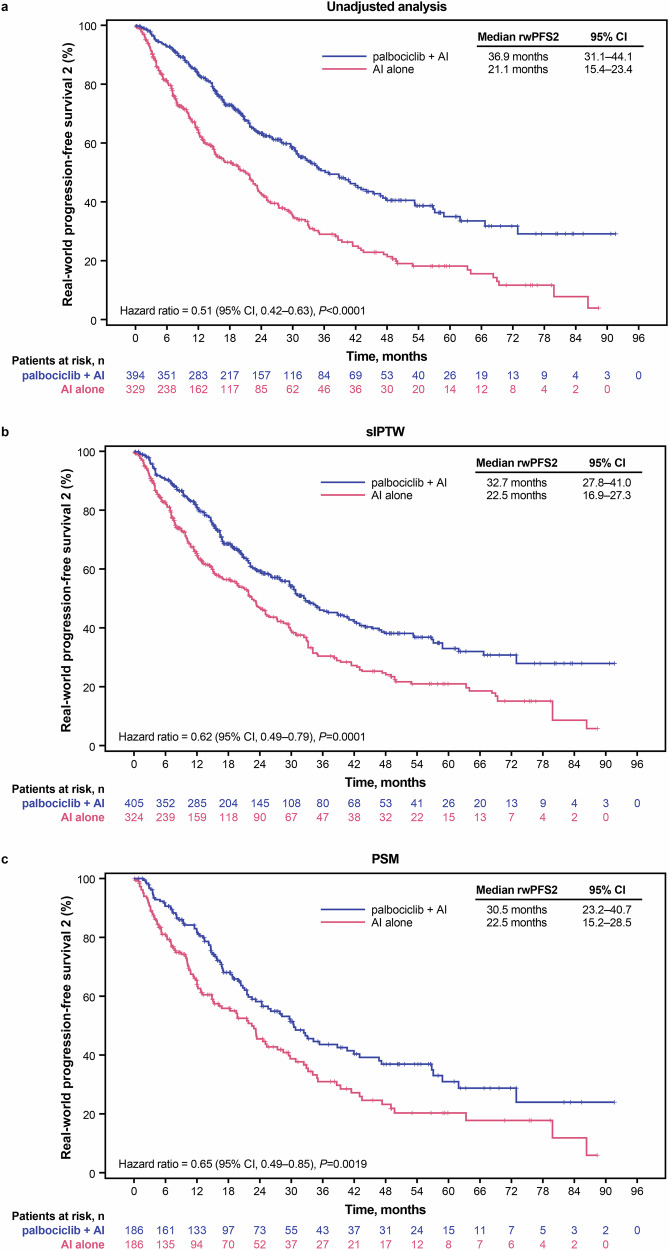
Kaplan–Meier curves of real-world progression-free survival 2. Graphical representation of rwPFS 2 in **a** the unadjusted population, **b** following sIPTW, and **c** following PSM. AI aromatase inhibitor, CI confidence interval, PAL palbociclib, PSM propensity score matching, rwPFS2 real-world progression-free survival 2, sIPTW stabilized inverse probability of treatment weighting.

Median rwPFS2 was significantly longer (36.9 months [95% CI, 31.1–44.1]) in the palbociclib plus AI group versus the AI alone group (21.1 months [95% CI, 15.4–23.4]; hazard ratio, 0.51 [95% CI, 0.42–0.63]; *P* < 0.0001; Fig. 4a). The results were similar after sIPTW (32.7 months [95% CI, 27.8–41.0] versus 22.5 months [95% CI, 16.9–27.3] in the palbociclib plus AI versus AI alone groups, respectively; hazard ratio, 0.62 [95% CI, 0.49–0.79]; *P* = 0.0001; Fig. 4b) and after PSM (30.5 months [95% CI, 23.2–40.7] versus 22.5 months [95% CI, 15.2–28.5], respectively; hazard ratio, 0.65 [95% CI, 0.49–0.85]; *P* = 0.0019; Fig. 4c).

### Subsequent treatment

In the unadjusted cohort, 177 of 394 patients (44.9%) treated with palbociclib plus AI received subsequent treatment: 77 (19.5%) received subsequent treatment with a CDK4/6 inhibitor; 37 (9.4%) received ET alone, 36 (9.1%) received chemotherapy, and 30 (7.6%) received another form of anticancer treatment (Table 2). Of the 235 of 329 patients (71.4%) in the AI alone group who received subsequent treatment, 120 (36.5%) received a CDK4/6 inhibitor, 80 (24.3%) received ET alone, 29 (8.8%) received chemotherapy, and 10 (3.0%) received other treatment (Table 2). Similar results were seen after the sIPTW and PSM analyses.

**Table 2:** Subsequent treatment for patients treated with 1 L palbociclib + AI versus AI alone

Subsequent treatment, *n* (%)	Unadjusted		sIPTW		PSM	
	1 L PAL + AI (*n* = 394)	1 L AI alone (*n* = 329)	1 L PAL + AI (*n* = 405)	1 L AI alone (*n* = 324)	1 L PAL + AI (*n* = 186)	1 L AI alone (*n* = 186)
**All treatments**	177 (44.9)	235 (71.4)	192 (47.4)	236 (72.8)	82 (44.1)	128 (68.8)
CDK4/6 inhibitor	77 (19.5)	120 (36.5)	77 (19.0)	123 (38.0)	33 (17.7)	73 (39.3)
Chemotherapy	36 (9.1)	29 (8.8)	35 (8.6)	29 (8.9)	18 (9.7)	13 (7.0)
Endocrine therapy alone	37 (9.4)	80 (24.3)	37 (9.2)	76 (23.5)	17 (9.1)	38 (20.4)
Other anticancer treatment	30 (7.6)	10 (3.0)	46 (11.4)	11 (3.4)	14 (7.5)	7 (3.8)

1L first-line, AI aromatase inhibitor, CDK4/6 cyclin-dependent kinase 4/6, PAL palbociclib, PSM propensity score matching, sIPTW stabilized inverse probability of treatment weighting.

**Fig. 5:**
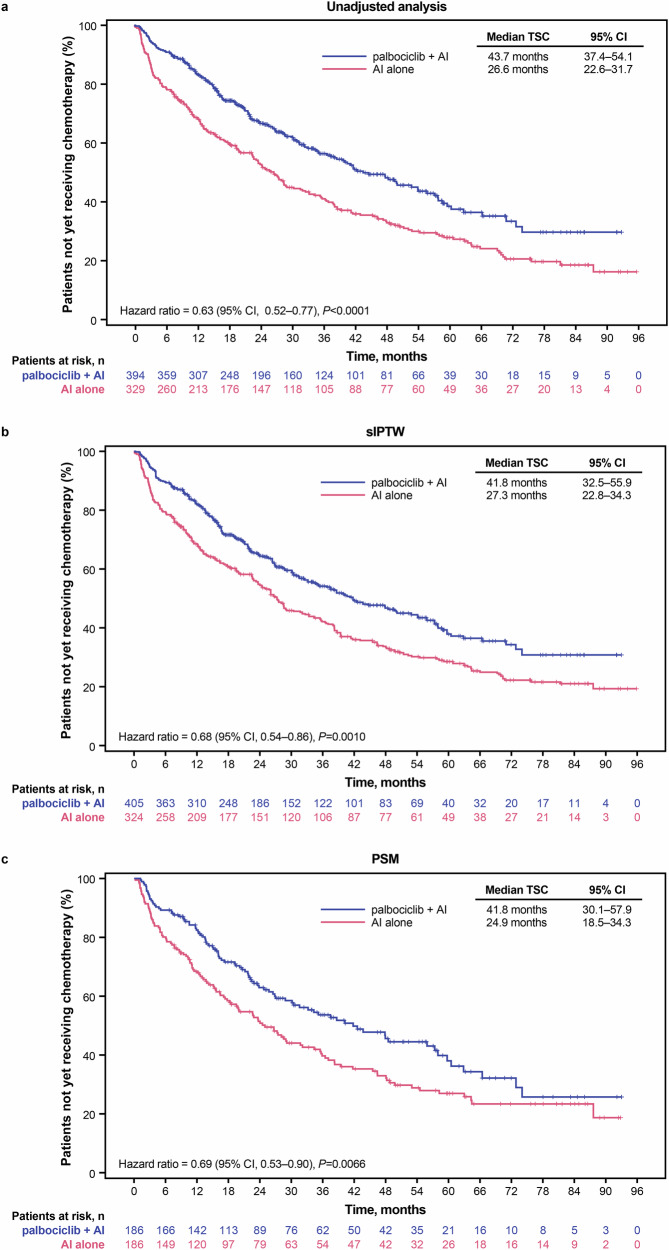
Kaplan–Meier curves of time to subsequent chemotherapy. Graphical representation of TSC in **a** the unadjusted population, **b** following sIPTW, and **c** following PSM. *AI* aromatase inhibitor, *CI* confidence interval, *PAL* palbociclib, *PSM* propensity score matching, *sIPTW* stabilized inverse probability of treatment weighting, *TSC* time to subsequent chemotherapy.

In the unadjusted analysis, median time to subsequent chemotherapy (TSC) was 43.7 months (95% CI, 37.4–54.1) in the patients treated with palbociclib plus AI versus 26.6 months (95% CI, 22.6–31.7) in the patients treated with AI alone (hazard ratio, 0.63 [95% CI, 0.52–0.77]; *P* < 0.0001; Fig. 5a). Similar results were seen after sIPTW (median TSC of 41.8 months versus 27.3 months respectively; hazard ratio, 0.68 [95% CI, 0.54–0.86]; *P* = 0.001; Fig. 5b) and after PSM (median TSC of 41.8 months versus 24.9 months, respectively; hazard ratio, 0.69 [95% CI, 0.53–0.90]; *P* = 0.0066; Fig. 5c).

## Discussion

Using the Flatiron Health longitudinal database, our analysis identified patients with HR+/HER2– MBC who received palbociclib plus AI or AI alone as first-line treatment and who resided in disadvantaged neighborhoods. Compared to an AI alone, palbociclib plus AI was associated with significantly prolonged median OS, rwPFS, rwPFS2, and TSC in the unadjusted analysis and after the sIPTW and PSM analyses.

Inequality in oncologic survival outcomes among various patient populations is a persistent challenge for care providers and policy makers. Patients with low SES are underrepresented in RCTs for cancer treatments^22^, thus creating data gaps in this context. As such, real-world data are necessary to determine the effectiveness of therapies for this vulnerable population. This study’s findings for patients living in disadvantaged neighborhoods and treated with first-line palbociclib are consistent with the PFS benefits demonstrated in RCT populations^2,3^ and observed in real-world studies^7,8^. The significant OS improvement with first-line palbociclib shown in this study is also in alignment with previous real-world studies^7,8^.

Even though it is recognized as guideline-recommended standard of care for HR+/HER2− MBC, combination CDK4/6 inhibitor and AI therapy has been deployed inequitably in patients in disadvantaged neighborhoods^13,14^. In a single institution study (*N* = 211), there were lower rates of CDK4/6 inhibitor use in urban versus suburban sites and a trend toward lower use for patients residing in low-income neighborhoods^14^. A more recent analysis using SEER data (*N* = 752) found that patients in counties with lower median household income (≤ $45 K USD) had significantly lower rates of treatment initiation with CDK4/6 inhibitors than those in counties with high median household income (> $60 K USD)^13^. Furthermore, in counties with a high percentage of dual Medicare–Medicaid coverage, a common proxy for social risk, the rate of treatment with CDK4/6 inhibitors was lower than in counties with a higher percentage of Medicare-only insurance^13^. Given the better clinical outcomes observed in our study with palbociclib plus an AI versus an AI alone, we speculate that the survival disparities for patients with HR+/HER2− MBC residing in areas with low SES may be driven in part by suboptimal administration of CDK4/6 inhibitors.

A variety of factors may contribute to underutilization of CDK4/6 inhibitors in patients living in disadvantaged neighborhoods. SDOH-related factors (e.g., lack of transportation, stable housing, and caregiver assistance) have been identified as key contributors to disparities in cancer management^21^. An observational study using the Flatiron Health database found that patients’ source of health insurance (Medicaid versus commercial provider) was associated with lower use of and reduced time on CDK4/6 inhibitors^23^. Financial toxicity associated with drug cost, treatment monitoring, and side-effect management may discourage patients and care providers from selecting optimal treatments^24,25^. Even after optimal treatment selection, financial stressors may negatively affect quality of life^26^ and contribute to medical non-compliance and/or poor treatment adherence^27,28^. CDK4/6 inhibitors may be insufficiently covered by insurance and there are time, cost, and transportation challenges inherent in cardiac, hematologic, and gastrointestinal monitoring, which is crucial for guideline-compliant care^29^. Cancer treatment decision-making is a complex process often affected by resource limitations^30^, but the evidence of improved clinical outcomes provided in this study may help more patients from disadvantaged neighborhoods to receive standard of care for MBC.

African American patients with cancer, a group that is disproportionally affected by SDOH^16^, have been shown to experience a greater risk of breast cancer-related death^31^. It has also been shown that Black/African American patients receive CDK4/6 inhibitors at a lower rate than White patients^23^. Indeed, population-level survival improvements since the introduction of CDK4/6 inhibitors have been driven by non-Hispanic White women without similar improvements for non-Hispanic Black and Hispanic women^32^. Real-world evidence suggests that African American patients with HR + /HER2 − MBC receive a significant survival benefit from palbociclib plus an AI compared with an AI alone^9^, so treatment disparities may represent opportunities to improve survival.

This study has some limitations. Because this was a retrospective observational study, it can only infer associations between treatments and outcomes, but not causality. Retrospective database analyses are also limited by the quality of the data captured and may be subject to incomplete, missing, or inaccurate data. Disease progression was not evaluated according to a predefined schedule, nor were standardized clinical trial assessments used, such as the Response Evaluation Criteria in Solid Tumors. As a result, rwPFS and rwPFS2 data relied on the treating physician’s interpretation of pathology reports and scan results. Additionally, treatments were not randomly assigned; instead, they were selected for each patient based on the treating physician’s clinical judgment and experience, which may have introduced selection bias. sIPTW and PSM were used to balance patient characteristics between treatment groups, but the effect of unmeasured potential confounders could not be adjusted for in these analyses. Our findings here inform only the comparison between palbociclib plus an AI versus an AI alone and do not assess comparisons among other CDK4/6 inhibitors in combinations with ET; future studies are necessary to assess the relative effectiveness of various CDK4/6 inhibitors in patients living in disadvantaged neighborhoods. Finally, the Flatiron Health database is limited to practices in the network in the United States, and hence these results may not be generalizable outside of this network. Nevertheless, our study fills an important gap in knowledge regarding palbociclib effectiveness in a vulnerable population in which there has been limited research and provides important evidence for stakeholders in the MBC treatment decision-making process.

In conclusion, this real-world database analysis demonstrates that first-line treatment with palbociclib plus AI, when compared with an AI alone, was associated with extended OS, rwPFS, rwPFS2, and TSC in patients with HR+/HER2– MBC living in disadvantaged neighborhoods. These findings support first-line palbociclib with ET as an effective option in this patient population.

## Methods

### Study design and data source

This was a retrospective, observational study of patient data captured in the United States nationwide Flatiron Health electronic health records-derived deidentified Enhanced Datamart for MBC (EDM; *N* = 33,448 at the time of data cutoff, December 2022). The Flatiron Health database contains patient-level structured and unstructured data, originating from ~280 cancer clinics (~ 800 sites of care), representing > 3 million actively treated cancer patients in the United States. Patients in the EDM who had HR+/HER2− MBC, were aged ≥ 18 years, and who started first-line treatment between February 2015 and June 2022 were randomly sampled (*n* = 5500). Of these, 5087 patients had first-line treatment data manually confirmed via abstraction (Fig. 1).

Neighborhood disadvantage was measured with the Yost index^33^, a composite measure of neighborhood social and economic vulnerability that has been well validated in previous cancer studies^19,34,35^. The Yost Index is reported as a percentile, with a higher percentage indicating higher socioeconomic status. The score for each census tract is calculated using seven variables: median household income, median home value, median gross rent, percentage of individuals living below 150% of the poverty line, percentage of individuals considered working class, percentage of individuals who are unemployed, and education index^19,35^.

To calculate the area-level socioeconomic status (SES) Index linked to patients in the Flatiron network, Flatiron uses a 5-year estimate of the variables listed above from the Census Bureau’s American Community Survey (2015–2019), the largest household survey in the United States. Yost Index quintiles, relative to the entire United States, were used to categorize neighborhood SES, with quintile 1 (i.e., the 20th percentile or lower) representing the lowest and quintile 5 (the 80th percentile or higher) representing the highest^33,35,36^. Disadvantaged neighborhoods were defined as Yost Index quintiles 1–2.

Patients included in the analysis had a diagnosis of HR+/HER2− MBC, 18 years of age or older, initiated palbociclib plus an AI or an AI alone as first-line therapy between February 2015 and June 2022 (index period), and lived in disadvantaged neighborhoods (Yost Index quintiles 1–2; Fig. 1). Patients were retrospectively assessed from start of palbociclib plus an AI or AI alone to December 2022 (data cutoff), death, or last medical activity, whichever came first.

This retrospective database analysis was conducted in accordance with the Guidelines for Good Pharmacoepidemiology Practice, Good Practices for Outcomes Research issued by the International Society for Pharmacoeconomics and Outcomes Research, Good Practices for Real-world Data Studies of Treatment and/or Comparative Effectiveness, and in accordance with the Declaration of Helsinki. As this study was retrospective, non-interventional, and used anonymized data, it was exempt from institutional review board approval and included a waiver of informed consent.

### Outcomes

The outcomes assessed were OS, rwPFS, rwPFS2, and TSC. OS was defined as the number of months from start of palbociclib plus AI or AI alone to death^7,8^. Date of death was a composite variable from three data sources: Social Security Death Index (SSDI), obituary data, and electronic health records-derived mortality data and has been previously validated against the National Death Index (NDI)^21,37^. rwPFS was defined as the number of months from the start of palbociclib plus AI or AI alone to death or disease progression, evaluated based on clinical assessment or radiographic scan/tissue biopsy^7,8^. rwPFS2 was defined as the number of months from the start of palbociclib plus AI or AI alone to disease progression on second-line therapy as determined by the treating physician, or death from any cause in either first-line or second-line, whichever occurred first^38^. TSC was defined as the length of time from the start of treatment to the beginning of subsequent chemotherapy or death from any cause, whichever came first^38^.

### Statistical analysis

Baseline demographics and disease characteristics were summarized using descriptive statistics. Median survival times for time-to-event endpoints were calculated using the Kaplan–Meier method and displayed graphically. The weighted Cox proportional hazards model was used to compute hazard ratios and corresponding 95% CIs. sIPTW (primary analysis) was used to balance baseline patient demographics and clinical characteristics, and 1:1 PSM was performed as a sensitivity analysis. Both sIPTW and PSM were based on propensity scores calculated using a multivariable logistic regression model. The variables included in the model were age group, sex, race/ethnicity, practice type, disease stage at initial diagnosis, Eastern Cooperative Oncology Group performance status, bone disease, visceral disease, interval from initial breast cancer diagnosis to MBC diagnosis, and number of metastatic sites^8^. The balance in these baseline characteristics was assessed using a standardized differences approach, with values ≥ 0.1 indicating a non-negligible imbalance. No imputation for missing values was performed. All data analyses were executed using statistical software SAS version 9.4 or later.

## Data Availability

Upon request, and subject to review, Pfizer will provide the data that support the findings of this study. Subject to certain criteria, conditions, and exceptions, Pfizer may also provide access to the related individual deidentified participant data. See https://www.pfizer.com/science/clinical-trials/trial-data-and-results for more information.
